# Response mechanism of hypocrellin colorants biosynthesis by *Shiraia bambusicola* to elicitor PB90

**DOI:** 10.1186/s13568-019-0867-5

**Published:** 2019-09-14

**Authors:** Wen Du, Chunlong Sun, Baogui Wang, Yanmei Wang, Bin Dong, Junhua Liu, Jiangbao Xia, Wenjun Xie, Jun Wang, Jingkuan Sun, Xuehong Liu, Hongguo Wang

**Affiliations:** 1 Shandong Provincial Engineering and Technology Research Center for Wild Plant Resources Development and Application of Yellow River Delta, College of Biological and Environmental Engineering, Binzhou University, Binzhou, China; 2 Shandong Provincial Key Laboratory of Eco-Environmental Science for Yellow River Delta, Binzhou University, Binzhou, China; 3 School of Biological and Environmental Engineering, Binzhou University, Binzhou, China

**Keywords:** Hypocrellin, *Shiraia bambusicola*, PB90, Nitric oxide, Hydrogen peroxide, Permeability

## Abstract

The valuable medicine *Shiraia bambusicola* P. Henn. and its major active substance hypocrellin exert unique curative effects on skin diseases, diabetes, and cancers. The wild *S. bambusicola* is endangered due to its harsh breeding conditions and long growth cycle. It is one of the effective ways to utilize the resources sustainably to produce hypocrellin by fermentation of *S. bambusicola*. PB90 is a protein elicitor isolated from *Phytophthora boehmeriae* to induce the useful metabolites production in fungi. In this work, PB90 was selected to promote the synthesis hypocrellin by *S. bambusicola*. To evaluate the effect of PB90 on *S. bambusicola*, it was found that the induced cells showed decreased biomass, increased cell wall permeability, rapid induction of secondary metabolites, and significant increase of some enzyme activities, which confirmed a strong activation of phenylalanine/flavonoid pathways. Studies on signal molecules and gene expression level in *S. bambusicola* treated with PB90 have found that hydrogen peroxide (H_2_O_2_) and nitric oxide (NO) are necessary signal molecules involved in the synthesis of hypocrellin in elicited cells, and increased their signal levels through mutual reaction. We have showed for the first time, the response mechanism of hypocrellin biosynthesis from *S. bambusicola* to PB90, which may be not only establish a theoretical foundation for the application of PB90 to the mass production of *S. bambusicola*, but can also motivate further research on the application of PB90 to the conservation and sustainable utilization of other medical fungi.

## Introduction

*Shiraia bambusicola* P. Henn. is a fungus that grows on bamboo branches and is mainly distributed in Zhejiang, Jiangsu, Anhui, Sichuan, and Guizhou provinces in China, as well as in Japan and Sri Lanka (Zhao and Chen 2018). *S. bambusicola* is a kind of precious medicinal fungus with mild nature and taste. This rare traditional Chinese medicine can promote blood circulation, remove blood stasis, dredge channels and collaterals, eliminate phlegm, relieve cough, and invigorate spleen to replenish qi (Zhao and Chen 2018; Du et al. 2012). Hypocrellin, the main active ingredient of *S. bambusicola*, is a photosensitive-pigment perylenequinone with anti-inflammatory, analgesic, antibacterial (Du et al. 2012), photosensitive tumor-cell-killing ability (Jia et al. 2017), anti-microvascular disease, and antidiabetes properties (Huang et al. 2016). Research on the nature and utilization of *S. bambusicola* relies mainly on field collection. The yield and quality are greatly influenced by climate, distribution, and harvest season. The direct use of *S. bambusicola* fermentation to produce hypocrellin is an ideal way to solve the growing shortage of crude natural-drug resources. Hypocrellin biosynthesis has the advantages of low investment, low equipment requirements, simple and safe operation, and mild reaction conditions. Most artificial cultures of *S. bambusicola* use synthetic medium and are cultured at 28 °C in liquid or solid medium. Most scholars have focused on the production of hypocrellin (Du et al. 2013; Lei et al. 2017; Sun et al. 2017) and anthraquinone compounds (Cai et al. 2008) by *S. bambusicola*, separating active substances and optimizing the culture medium composition and culture conditions. In addition to the common optimization process, hypocrellin synthesis from *S. bambusicola* has been found to be promoted by the elicitor *Trametes* sp. (Du et al. 2013), surfactant Triton X-100 (Lei et al. 2017), ultrasonic wave (Sun et al. 2017), and light (Sun et al. 2018). Through these steps, hypocrellin yield has been found to increase several times to tens of times. Indeed, different components of the medium and environmental conditions greatly influence hypocrellin synthesis from *S. bambusicola*. Nevertheless, many problems in hypocrellin biosynthesis are still encountered, for example, optimize contents are repeated (Du et al. 2013; Lei et al. 2017; Sun et al. 2017), the hypocrellin synthesis mechanism is unknown (Gao et al. 2018), the status of strains used is unclear, and the active metabolite content is low (Du et al. 2013; Sun et al. 2017). This series of problems hinders the commercial production of hypocrellin and can be solved by exploring ways to improve hypocrellin yield.

Most secondary metabolites are not directly involved in the growth and development of fungus. Thus, secondary metabolite synthesis is strictly controlled in fungus growing under normal conditions (Xu et al. 2011). In cell culture, the synthesis of many secondary metabolites is part of the cellular defense response to pathogen attack (Ishihara et al. 2011). Therefore, the use of fungal elicitors is one of the most effective strategies for increasing the productivity of useful secondary metabolites in cell culture (Zhao et al. 2015). Specific fungal elicitors can increase the accumulation of secondary metabolites in plant cells and have thus been successfully applied in plant-cell-suspension cultures with potential for commercialization (Simic et al. 2015). Indeed, the use of elicitors to promote the rapid and large-scale synthesis of useful secondary products by fungal cells has become a new important method (Du et al. 2013). PB90 is a protein elicitor (molecular weight = 90 kDa) isolated from *Phytophthora boehmeriae* (Wang et al. 2003). The infiltration of PB90 into tobacco leaves reportedly induced hypersensitive responses and systemic acquired resistance (Zhang et al. 2007). In tobacco and Chinese cabbage, PB90 treatment strongly enhanced the pathogen-related accumulation of PR-1 and the expression of chitinase (Li et al. 2006). Treatment of PB90 stimulated hypericin production and hydrogen peroxide (H_2_O_2_) generation in *Hypericum perforatum* cells and H_2_O_2_ was essential for PB90-induced hypericin production (Xu et al. 2011). These results suggested that PB90 may induce plant defense responses and accumulation of secondary metabolites, and so on. However, few researchers have studied the effect of PB90 on fungal metabolites, and no one has studied the effect of PB90 on the secondary metabolites of *S. bambusicola*. The accumulation of many secondary metabolites is widely believed to be a defense mechanism of cellular against pathogenic attacks (Zhai et al. 2016). Thus, obtaining useful elicitors from microorganisms can effectively improve the productivity of useful secondary metabolites in cell cultures. To examine the effect of PB90 on the secondary metabolites of cultured fungal cells, we measured hypocrellin yield in *S. bambusicola* after PB90 treatment. Results showed that PB90 was an effective elicitor of increasing hypocrellin yield in *S. bambusicola*. The effects on *S. bambusicola* biomass, cell wall,metabolites, signal molecules, and related genes were also examined.

## Materials and methods

### Strains, chemicals and culture medium

*Shiraia bambusicola* BZ-16X1 was collected and identified by the Institute of Fungal Resources, Binzhou University, China. The strain of *S*. *bambusicola* was stored in the China Center for Type Culture Collection, and its preservation number was CCTCC NO: M209141. *S*. *bambusicola* was cultured in potato dextrose agar (PDA) for 7 days and transferred into a 500 mL triangular flask containing 150 mL of basal medium for shaking culture at 26 °C and 120 r/min. The basal medium was made from 15 g/L glucose, 1.5 g/L sodium nitrate, 1.0 g/L KH_2_PO_4_, 0.5 g/L MgSO_4_·7H_2_O, 0.1 g/L Na_2_HPO_4_·5H_2_O, 0.01 g/L CaCl_2_, 1 mg g/L FeSO_4_·7H_2_O, 2 mg CuSO_4_·5H_2_O. In this study, spores and mycelia were scraped from the PDA tablet surface and beaten using sterile glass beads, diluted 10^7^ spores/mL with aquae sterilisata, inoculated in a triangular flask containing the fermentation medium, shaken at 26 °C and 120 r/min, and cultured for 4 days. The mycelium pellets were placed into a sterile mixer and centrifuged for 10 s at 3000 rpm, forming the homogeneous mycelia for vaccination. The homogeneous *S*. *bambusicola* was added to the optimized and basal media by 20% of the inoculation amount. 5 nmol/L PB90 was added of the inoculation amount to the *S*. *bambusicola*, medium cultured for 3 days, and recultured for 1–9 days. Carboxy-2-phenyl-4,4,5,5-tetramethylimidazoline-1-oxyl-3-oxide(cPITO), superoxide dismutase (SOD) and catalase (CAT) were purchased from Sigma-Aldrich (St. Louis, MO, USA). A protein with a molecular weight of 90 KD was isolated from the culture solution of *P. boehmeriae* (Xu et al. 2011) as a fungal inducer (PB90). All other chemicals and reagents were analytical-grade.

### Determining biomass

The fermented mash was filtered through a funnel, with the filtrate removed. The collected thalli were washed with distilled water, and after the removal of the supernatant, the thalli were dried to a constant weight at 35 °C and weighed.

### Observation of the PB90 action on the membrane structure

Different volume of PD medium, SNPs solutions, and *S*. *bambusicola* cells were added to 10 mL cultures resulting in final concentration of 50 μg/mL. The control received vehicle solvent only. Three-day-old cells treated with 5 nmol/L PB90, the cultures were centrifuged at 3 days after PB90 was added and the supernatants discarded for observation by transmission electron microscopy (TEM, Hitachi, Japan) and scanning electron microscopy (SEM, Hitachi, Japan).

### Observation of the membrane vesicles structure

The membrane isolation and vesicle preparation were performed using the method of Li et al. (2010) with some modifications. Buffer A [50 mM Tris–HCl buffer (pH 8.0) containing 2 mM sodium dithionite] was employed throughout. The cell-free extracts of *S*. *bambusicola* were prepared by suspending 5 g (wet weight) of frozen cells in 50 mL buffer A. The cell suspensions were then sonicated in an ice bath, and the cell breakage was monitored by examining the cells with a microscope. The unbroken cells were removed by centrifugation at 10,000 rpm for 15 min, and the crude extract was centrifuged at 20,000 rpm for 2 h. The resulting pellets contained the cell membranes, whereas the cytoplasmic proteins remained in the supernatants. The membrane fractions were resuspended in buffer A to allow the spontaneous formation of vesicles, which were confirmed by TEM. Fifty milliliters of membrane vesicles was treated with 5 nmol/L PB90 for 1 h, and the control received vehicle solvent only. The membrane vesicles exposed and unexposed to PB90 were observed via TEM.

### Determining the total phenolics content in mycelia

The scraped mycelia were extracted for 10 min with the alcohol-acetone solution (1:1) in water bath by ultrasonic method at room temperature and centrifuged at 4800 r/min, and the supernatant was collected and sampled with the right amount of Folin–Ciocalteu reagents and 20% sodium carbonate solution, evenly mixed and placed quietly for 40 min. The absorbance was determined with the wavelength of 725 nm. The results were expressed as milligrams of gallic acid equivalents (GAE) per gram of dry mass (mg GAE/g DM) (Du et al. 2017).

### Determining the total flavonoids content in mycelia

Ethanol solution (60%) was added to the scraped mycelia by the solid–liquid ratio of 1:15, and the mycelia were extracted for 1 h at 80 °C. The filtrate after the repeated extraction and filtration was added to the chromatographic column of macroporous resin AB-8 for adsorption, and with impurities (such as pigments) scoured off with distilled water until a colorless effluent was discharged, and eluted with 80% ethanol solution. Then, a concentrated eluent was collected and sampled. The sampled eluent with 5% NaNO_2_, 10% Al(NO_3_)_3_, and 1 mol/L NaOH added was shaken up and placed for 30 min, with the absorbance determined with the wavelength of 510 nm. The results were expressed as milligrams of catechin equivalents (CE) per gram of dry mass (mg CE/g DM) (Chai et al. 2013).

### Determination of enzyme activity

The activity of phenylalanine ammonia-lyase (PAL) and chalcone isomerase (CHI) was determined by reference to Du et al. (2017).

### Determination of gene transcription lever

According to the method of Li et al. (2019), the method has been improved slightly. The culture of *S. bambusicola* and PB90 was compared with that of *S. bambusicola* with equal amount of aseptic water. RNA extraction in accordance with the Qiagen Rneasy Plant Mini Kit with Nanodrop and agarose gel electrophoresis to detect RNA concentration and quality, of after testing qualified RNA samples stored in − 80 °C refrigerator. The synthesis of cDNA reference TaKaRa company Prime Script^®^ RT reagent Kit briefly operating instructions. Gene expression levels were detected by real-time quantitative PCR (bio-rad, Hercules, USA). The primers required for PCR reaction are shown in Additional file 1: Table S1. The 18S gene was the internal standard, and the relative gene expression levels were calculated by using 2^−△△*Ct*^ and repeated five times.

## Results

### Effects of PB90 on *S. bambusicola* biomass and hypocrellin production

**Fig. 1:**
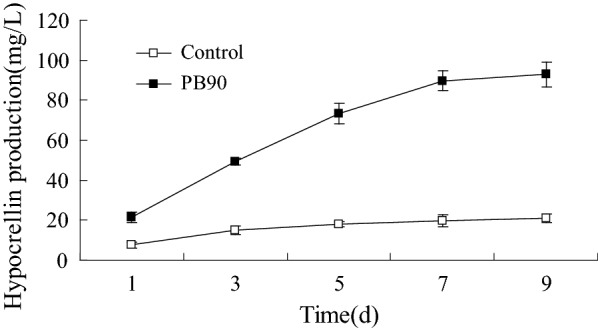
Effects of PB90 on hypocrellin production. Tree-day-old cells treated with 5 nmol/L PB90 were harvested as the time indicated in the figure. The control received vehicle solvent only. The results shown are the average of five independent experiments. Bars represented standard errors

The effects of PB90 on the yield of hypocrellin in cultured cells of *S. bambusicola* were evaluated, and results are shown in Fig. 1. Experimental results showed that *S. bambusicola* had rapid and strong reactions to the application of PB90 by increasing hypocrellin biosynthesis. Compared with the control group, hypocrellin yield significantly increased by 2.5 to 4.5 times throughout the entire induction period. That the production of hypocrellin was significantly increased in *S. bambusicola* cells treated with PB90, and the yield was up to 278.71 mg/L. The elicitor PB90 can be used as an effective elicitor for obtaining valuable bioactive compounds from *S. bambusicola* cells.

In addition, 9 days after PB90 treatment, browning and cell aggregation of cultured cells of *S. bambusicola* were also observed. The fresh weight of the biomass of induced and cultured *S. bambusicola* was always lower than or equal to the control value (Additional file 1: Fig. S1). Until the 5th day after induction, the biomass of induced cells still did not significantly differ from that of the control group. From day 7 to day 9, the fresh biomass yield of the induced cells decreased by about 20–33% compared with that of the control group. Obviously, PB90 was negatively correlated with the biomass of cell culture of *S. bambusicola*, we speculated that PB90 treatment increased hypocrellin yield by metabolic flux shunting.

### Effects of PB90 on the morphology of *S. bambusicola*

**Fig. 2:**
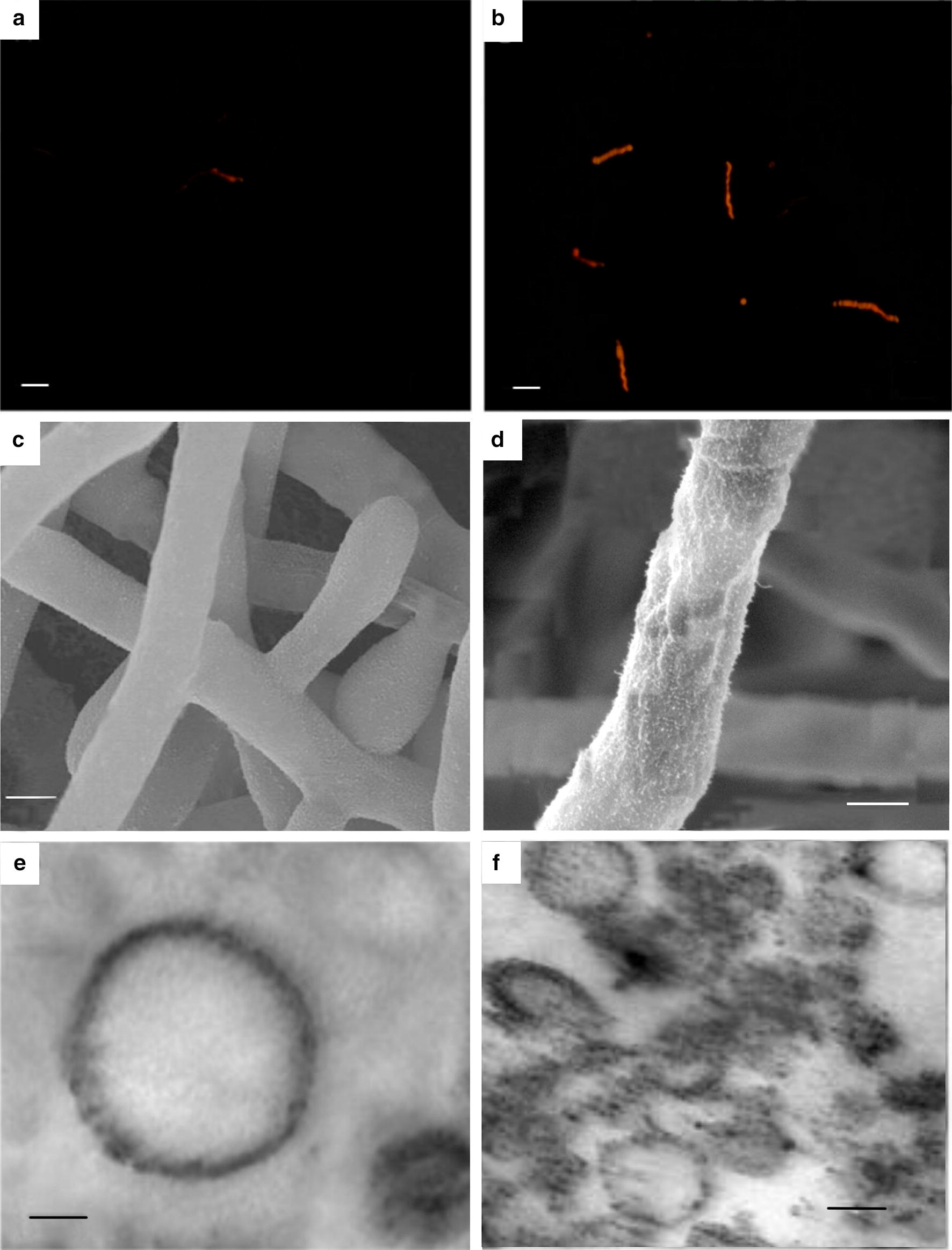
Microscope images of *S. bambusicola* treated with PB90. **a** *S. bambusicola* observed by fluorescence microscope, bar = 20 μm. **b** Action of 5 nmol/L PB90 on *S. bambusicola* by fluorescence microscope, bar = 20 μm. **c** *S. bambusicola* observed by SEM, bar = 4 μm. **d** Action of 5 nmol/L PB90 on *S. bambusicola* observed by SEM, bar = 2 μm. **e** *S. bambusicola* vesicles observed by TEM, bar = 80 nm. **f** Action of 5 nmol/L PB90 on *S. bambusicola* vesicles observed by TEM, bar = 200 nm

The effect of PB90 on the morphology of *S. bambusicola* was observed by microscope (Fig. 2). Fluorescence microscopy showed that after the treatment of PB90, the pyridine iodide stain entered the cell and combined with the nucleic acid to fluoresce. This indicates that PB90 can destroy the permeability of the cell membrane of *S. bambusicola*. Through SEM observation, it was found that PB90 could damage the cell membrane, morphological changes on the cell surface were observed, and the control cells were plump. After the effect of PB90, the membrane vesicles of *S. bambusicola* were extracted and observed by TEM, and it was found that some membrane vesicles were distorted and could no longer form into globules, indicating that PB90 treatment acted on the vesicles in the cell membrane during the process of promoting the hypocrellin synthesis.

### Effects of PB90 on the metabolites and enzymes of the phenylpropanoid biosynthetic pathway

**Fig. 3:**
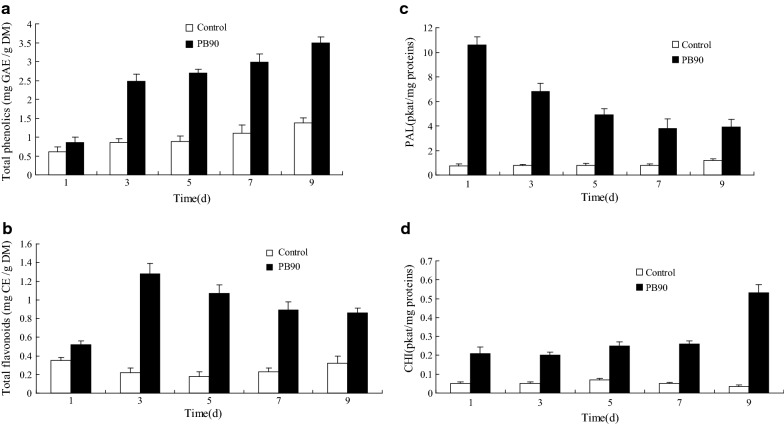
Effects of PB90 on total phenolics (**a**), total flavonoids (**b**), the activity of PAL (**c**) and CHI (**d**) production. Tree-day-old cells treated with 5 nmol/L PB90 were harvested as the time indicated in the figure. The control received vehicle solvent only. The results shown are the average of five independent experiments. Bars represented standard errors

The biological infection of plant tissues most commonly causes changes in the metabolic activity of the phenylpropanoid biosynthesis pathway. Accordingly, our research group also detected many metabolites and enzymes related to this pathway. The effects of PB90-induced culture on total phenolics accumulation in the culture medium of *S. bambusicola* are shown in Fig. 3a. Compared with the control (without PB90), the total phenolics content of *S. bambusicola* significantly increased from day 1 to day 9. On the 3rd day after culture induction, total phenolics yield rapidly increased to 2.9 times, it maintained a steady growth after a long induction period, the phenol content increased to 2.5 times on day 9 of induction. Meanwhile, the total flavonoids yield in the cultured cells significantly increased from day 3 to day 9 compared with the control group (Fig. 3b). On day 3, the total flavonoids yield increased by about 5.8 times and decreased slightly, but the overall increase was still significant compared with the control group. Studies have shown that PB90-induced cultures can activate an increase in hypocrellin production and be an effective strategy to enhance different active metabolites.

We further examined the activities of PAL and CHI, the key enzymes of the phenylpropanoid/flavonoid compound pathway, by the PB90-induced cultures. The activities of PAL and CHI (Fig. 3c, d) were found to be strongly induced in the cultured cells, and induction levels depended on induction culture time. After induction, the PAL activity of cell suspension increased and peaked on the first day by about 14 times that of the control group. Afterwards, PAL activity gradually decreased but still significantly increased, becoming about three times higher than that of the control group until day 9. The CHI enzyme activity of the cultured cells was enhanced during the culture-induction period. By day 9, the PB90-induced culture significantly stimulated CHI activity to increase by about 15 times compared with the control sample. Analysis also revealed that the yield of total phenols, total flavonoids and hypocrellin in the cells treated with PB90 had a significant and positive correlation with PAL and CHI enzyme activities.

### Effect of PB90 induced hypocrellin biosynthesis on signaling molecules activity

#### Effect of PB90 induced hypocrellin biosynthesis on signaling molecules

**Fig. 4:**
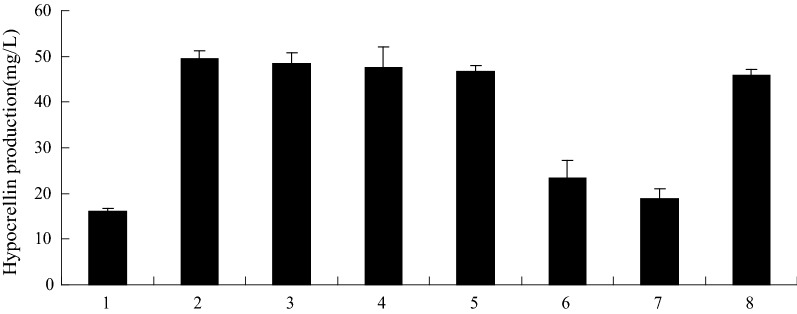
Effects of different quenchers on PB90-triggered hypocrellin production. Three-day-old cells treated with 10 mmol/L glycerol, 10 mmol/L histidine, 200 units/mL SOD, 200 units/mL CAT, 0.5 mmol/L cPITO, 150 mg/L verapamil and 5 nmol/L PB90, hypocrellin production were measured at 3 days after PB90 was added. The quenchers were added 30 min before PB90 treatment. The control received vehicle solvent only. Values were means of five independent experiments. Bars represented standard errors. (1) Control; (2) PB90; (3) PB90 + glycerol; (4) PB90 + histidine; 5. PB90 + SOD; (6) PB90 + CAT; (7) PB90 + cPITO; (8) PB90 + verapamil

The inducible signal transduction process is a complex network in which various signals are integrated into DNA transcription factors, ultimately leading to defensive reactions and metabolic pathways that induce secondary metabolites. Nitric oxide (NO), salicylic acid (SA) and reactive oxygen species (ROS) are important signaling molecules that regulate cell resistance reactions and play an important role in the process of secondary metabolism (Yin et al. 2016). The accumulation of secondary metabolites by fungal elicitors is a defensive reaction. To investigate the cells involved in mediating PB90 to induce hypocrellin synthetic signal molecule and pathways, understand the role of NO, Ca^2+^, hydroxyl radicals, H_2_O_2_, superoxide anion radical, and singlet oxygen in induction culture, various active oxygen scavengers and quenching agents were added to the culture solution. For example, glycerin (10 mM) can be used as hydroxyl radical quenching agent, histidine (10 mM) as a singlet oxygen quencher, superoxide dismutase (SOD, 160 units/mL) as superoxide anion radical quencher, and CAT (160 units/mL) as H_2_O_2_ quencher (Ishikawa et al. 2010; Du et al. 2017), cPITO (50 mm mol/L) can be used as NO specific quenching agent, and verapamil (150 mg/L) can be used as Ca^2+^ scavenger (Park et al. 2018). Results as shown in Fig. 4, CAT and cPITO can significantly reduce the yield of hypocrellin after induction culture, while glycerol, histidine, SOD and verapamil were no significant influence on the yield of hypocrellin. The results showed that the induced culture of elicitor PB90 was closely related to the amount of H_2_O_2_ and NO. In addition, we also tested the increase of H_2_O_2_ and NO by 5.3 times and 2.5 times, respectively, after 24 h treatment by PB90, compared with *S. bambusicola* not treatment by PB90.

#### Synergistic effects of NO and H_2_O_2_ in PB90-induced hypocrellin biosynthesis

**Fig. 5:**
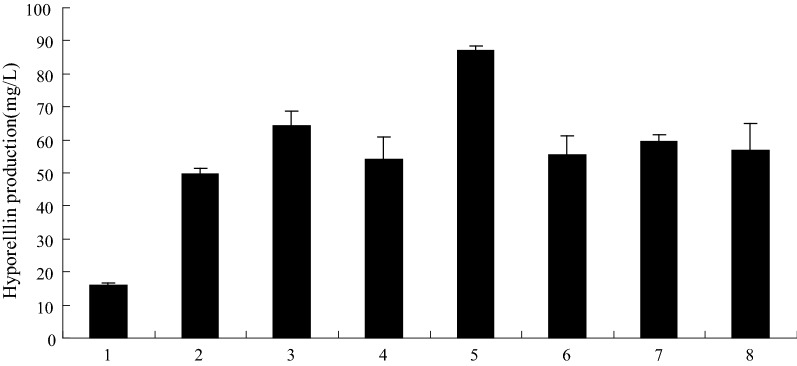
Synergistic effect of H_2_O_2_ and NO on the synthesis and accumulation of hypocrellin. Three-day-old cells treated with 5 nmol/L PB90, 5 mmol/L SNP (about 5 mol/L NO), 15 μmol/L H_2_O_2_, 0.5 mmol/L cPITO and 0.5 mmol/L CAT, hypocrellin production were measured at 3 days after PB90 was added. The adding time of cPITO and CAT was 30 min before NO and H_2_O_2_ treatment. The control received vehicle solvent only. The results shown are the average of five independent experiments. Bars represented standard errors. (1) Control; (2) PB90; (3) PB90 + SNP; (4) PB90 + H_2_O_2_; (5) PB90 + SNP + H_2_O_2_; (6) PB90 + SNP + H_2_O_2_ + cPITO; (7) PB90 + SNP + H_2_O_2_ + CAT; (8) PB90 + SNP + CAT

Figure 5 shows that NO and H_2_O_2_ are the necessary conditions in the mediation of synthesis and accumulation of hypocrellin in *S. bambusicola*. To examine the effects of NO and H_2_O_2_ on the synthesis and accumulation of hypocrellin in *S. bambusicola* cells, the influence of exogenous NO and H_2_O_2_ on hypocrellin yield was determined. Sodium nitroprusside (SNP), which is the donor of NO, can significantly increase the yield of hypocrellin in *S. bambusicola*, and the promotion of SNP on the synthesis and accumulation of hypocrellin can be inhibited by the NO-specific quencher (Fig. 5). However, other decomposition by-products of SNP, such as sodium ferricyanide, have no significant effect on hypocrellin yield (data not reported). Therefore, the results of this experiment indicate that NO can promote the synthesis and accumulation of hypocrellin in *S. bambusicola*. The hypocrellin yield in the cells treated with exogenous H_2_O_2_ was not significantly different from the yield of those in the control, thereby indicating that H_2_O_2_ itself is not a sufficient factor to induce the synthesis and accumulation of hypocrellin. However, even if H_2_O_2_ itself cannot promote the production of hypocrellin in cells, the hypocrellin yield in *S. bambusicola* under joint treatment of H_2_O_2_ and NO was significantly higher than that obtained with NO alone (Fig. 5). Thus, H_2_O_2_ and NO have synergistic induction effect on the synthesis and accumulation of hypocrellin.

### Effect of PB90 induced hypocrellin biosynthesis on some genes expression

**Fig. 6:**
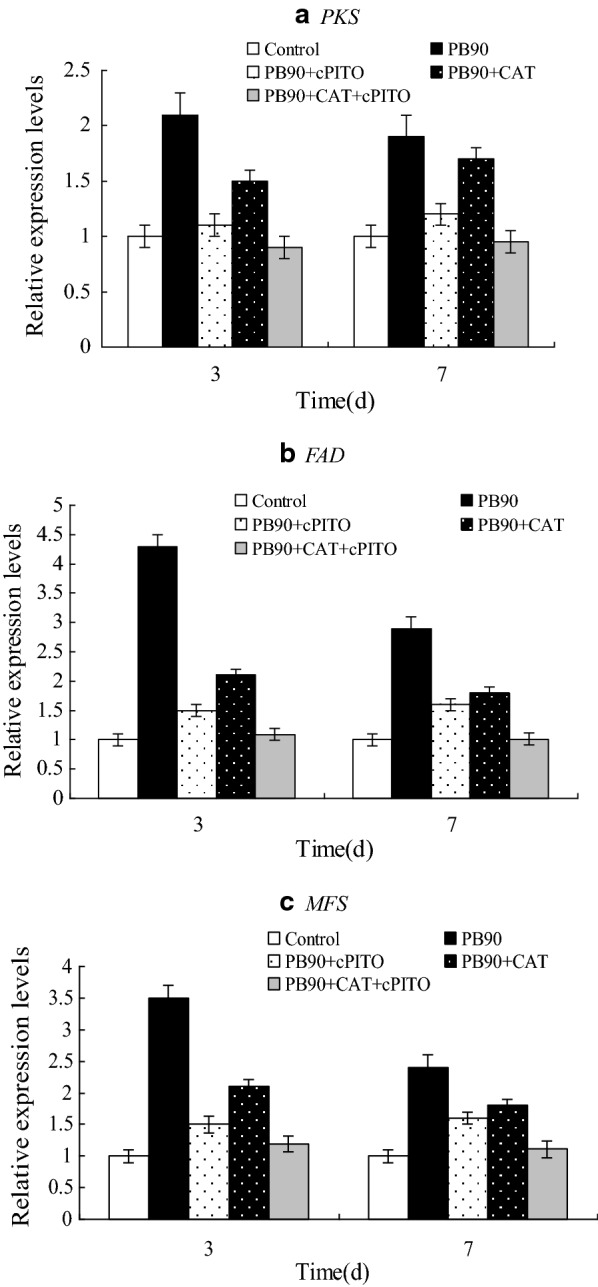
Effects of PB90 on some genes expression of *S*. *bambusicola*. *PKS*: polyketide synthase (**a**), *FAD*: FAD/FMN-dependent oxidoreductase (**b**), *MFS*: major facilitator superfamily (**c**). Three-day-old cells treated with 5 nmol/L PB90, hypocrellin production were measured at 3 days after PB90 was added. 0.5 mmol/L cPITO and 0.5 mmol/L CAT were added to 3-day-old cells respectively. The adding time of cPITO and CAT was 30 min before PB90 treatment. The control received vehicle solvent only. The results shown are the average of five independent experiments. Bars represented standard errors

The signal transduction of fungal elicitors can induce the synthesis of new enzymes and can activate new metabolic pathways, thereby benefiting the synthesis of secondary metabolites possibly by increasing the number of key enzymes that synthesize secondary metabolites. We examined polyketide synthase (*PKS*), which is related to the synthesis of hypocrellin, FAD/FMN-dependent oxidoreductase (*FAD*) related to redox state, and promotion of major facilitator superfamily transporter (*MFS*) associated with signal transduction. The genes were detected by real-time quantitative PCR. Under the action of PB90, CAT and cPITO expressed three differentially expressed genes in *S. bambusicola*, as shown in Fig. 6.

The expression levels of these genes were up-regulated under the action of PB90, thereby indicating that the expression of *PKS*, *FAD*, and *MFS* genes were positively correlated with the synthesized amount of hypocrellin induced by PB90 in *S. bambusicola*. After adding CAT and cPITO, the expression levels of *PKS*, *FAD*, and *MFS* genes significantly decreased. These levels were almost equal to the levels in the control. When CAT and cPITO were added separately, the expression levels of *PKS*, *FAD*, and *MFS* genes were significantly lower than those of PB90 group, but not much lower than those when CAT and cPITO were added at the same time. This further confirmed the synergistic effects of H_2_O_2_ and NO on PB90-induced accumulation of hypocrellin.

## Discussion

Hypocrellin is a natural product of *S. bambusicola* and has a unique therapeutic effect on patients with diabetes (Huang et al. 2016). In addition to antidiabetic properties, hypocrellin can induce apoptosis and radiosensitize tumor cells. It has anti-inflammatory and phototherapy effects on skin diseases (Zhang et al. 2016; Jia et al. 2017). Fungal cell culture produces active metabolites with various complex structures due to the enormous commercial value of active metabolites, limited supply, or endangered status of parental species, and this has been the focus of researchers (Knapp and Kovács 2016; Zhao et al. 2016). Cell culture of *S. bambusicola* provides an alternative for hypocrellin production. Most secondary metabolites are not directly involved in the growth and development of fungus. Thus, secondary metabolite synthesis is strictly controlled in fungus growing under normal conditions (Xu et al. 2011). In cell culture, the synthesis of many secondary metabolites is part of the cellular defense response to pathogen attack (Ishihara et al. 2011). Therefore, the use of fungal elicitors is one of the most effective strategies for increasing the productivity of useful secondary metabolites in cell culture (Zhao et al. 2015). Inducers prepared in pathogens can be used to improve secondary metabolites of cells. PB90 is a novel protein inducer secreted by *P. boehmeriae*. PB90 treatment reportedly triggered a highly sensitive reaction in tobacco plants (Li et al. 2006). In addition to hypersensitivity reactions, secondary metabolite accumulation is another common response of cells to multiple biotic and abiotic stresses. However, few researchers have studied the effect of PB90 on fungal metabolites, and no one has studied the effect of PB90 on the secondary metabolites of *S. bambusicola*. Results of our study showed that the production of hypocrellin was significantly increased in *S. bambusicola* cells treated with PB90. Production reached approximately 2.5-fold within 24 h after treatment, thereby indicating that PB90 can induce the production of hypocrellin in *S. bambusicola* cells. Therefore, it is suggested that PB90 might be a new potential inducer that can improve secondary metabolite production of fungal cells.

The induction effect of the elicitor on cell growth depends on the type, concentration, and cell growth stage of the elicitor at the time of challenge application (Simic et al. 2015). Polysaccharide extracts of the exogenous fungal mycelium negatively affect the biomass of *S. bambusicola* cell culture. In the mixed culture of *S. bambusicola* and bamboo endophytic fungus *Phoma* sp. BZJ6, the same results were also found (Du et al. 2017). However, the crude polysaccharide elicitor of endophytic fungus *Trametes* sp. TT1 can stimulate the production of the biomass of *S. bambusicola* cells (Du et al. 2013). Some bacterial crude polysaccharide elicitors did not show any stimulating effect on cell growth after treatment (Du 2010). These findings suggest that the response of *S. bambusicola* cells to specific elicitors may vary depending on the cell line and culture conditions. Inhibition of cell growth and survival after induction is caused by a metabolic flux shunt, i.e., the activation of secondary metabolism suppresses the primary metabolic activity (Sivakumar and Paek 2005). Indeed, the induction of cell growth and the production of secondary metabolites are often negatively correlated, and the elicitor PB90 can be used as an effective elicitor for obtaining valuable bioactive compounds from *S. bambusicola* cells. Observation of elicitor PB90 role of *S. bambusicola*, found that the action of PB90 causes cell morphological changes, can increase the permeability of the cell wall, reduce the stability of vesicles in the cell wall, thus increasing the accumulation of hypocrellin. The result is similar to some studies, for example, Triton X-100 treatment of *S. bambusicola* enhanced hypocrellin production was mainly due to both elicited biosynthesis in mycelium and the increased membrane permeability for hypocrellin release (Lei et al. 2017). After the tobacco was treated with PB90, DAPI staining observed the morphological characteristics of chromatin agglutination and nuclear disintegration in the cells (Ji et al. 2005).

PB90 caused a large accumulation of total phenolics and flavonoids in the cells induced by *S. bambusicola*. When treated with fungal elicitors *Fusarium oxysporum*, *Phoma exigua* and *Botrytis cinerea*, the production of naphtodianthrones and phenylpropanoids in the cell suspension of *H. perforatum* were also stimulated (Simic et al. 2015). The accumulation of total phenolics and flavonoids by *H. perforatum* under the induction of the fungus *Nomuraea rileyi* shows that these active compounds are the inducing part of the cellular defense response (Meirelles et al. 2013). Therefore, the increase in phenylpropanoid production may be due to the activation of plant cell defense pathways. Extracellular products are released from the cells. As observed in this study, the specific effects of fungal inducers are most likely related to fungal interactions and the complexity of inducing signal transduction, thereby leading to cellular defense responses (Garrido-Gala et al. 2014). The current study indicates that PB90 can induce the enzyme activity of PAL and CHI. Such rapid PAL activation may be related to the fact that PB90 is a protein from *P. boehmeriae*, which can trigger early plant defense response (Chen et al. 2017). The research results support the increase in PAL activity in different cell cultures induced by fungus (Ganapathy et al. 2016). Even if PAL is a key regulatory enzyme that leads to the formation of a wide range of phenylpropanoid metabolites (Sabela et al. 2016), CHI is necessary for the biosynthesis of flavonoids (Ververidis et al. 2007). The increase in flavonoid accumulation in cucumber plants under the induction of the fungal pathogen *Sphaeroteca fuliginea* indicates the significance of these phenolic compounds in plant defense systems (Fofana et al. 2002). In our previous study on mixed cultures of *Phoma* sp. BZJ6 and *S. bambusicola*, synergistic induction of PAL and CHI was associated with the significantly higher laccase activity in cells after fungal mixed culture (Du et al. 2017). The most effective way to use the data is to apply it to liquid culture in the process of making better use of the cells, or to insert the elicitors into the cells cultured in solid medium to resolve the difficulties that may be encountered. Therefore, promoting the infiltration of fungal elicitors in suspension cells can effectively trigger the enzyme activity of PAL and CHI. If such a consequence exists in cells treated with PB90, then changes in phenol levels may be a response to changes in the enzyme.

Phenylpropanoid biosynthesis is one of the most common metabolic activities caused by pathogenic infection in cells or cultured cell treatment with different elicitors. The strong and rapid stimulating effect of fungal elicitors on plant secondary metabolite accumulation has been extensively investigated (Li et al. 2014; Singh et al. 2017). A simultaneous increase in the production of phenylpropanoid and hypocrellin and the enzyme activity of PAL and CHI indicates that PB90 is an effective strategy to enhance different phenolic compounds. However, the signal transduction mechanism of fungal elicitors that mediate the synthesis of fungal secondary metabolites is poorly understood. NO and H_2_O_2_ are two common signaling molecules in cells. Results revealed that NO and H_2_O_2_ were the signaling molecules necessary for PB90-induced synthesis of hypocrellin. The hypocrellin production of *S. bambusicola* treated with the combination of NO and H_2_O_2_ was obviously higher than that under the single NO treatment, suggesting that NO and H_2_O_2_ could elicit synergistic effects through signal cooperation in hypocrellin-mediated synthesis. Previous research showed that *PKS* can be used to catalyze the early step of the condensation reaction of acetyl-CoA and malonyl-CoA subunits (Sun et al. 2017). The claisen-type cyclization of hypocrellin aromatic ring can be possibly catalyzed by PKS-type thioesterase, the expression level of *PKS* was significantly increased under PB90 in our current study. The expression levels of *FAD* gene greatly increased with the interaction of PB90 possibly because fungal cells produced more active oxygen when they interacted with the elicitors. As a result, these cells evolved into their higher antioxidant state (Nowogórska and Patykowski 2015). Therefore, the normal redox state of cells should be maintained (de Simone et al. 2017). Oxidoreductase can reduce aldehydes or ketones to form various alcohols or amine compounds. The superfamily of *MFS* transporters are composed of many members and widely found in lower and higher organisms. Nearly a quarter of the transporters encoded in the microbial genome belong to this superfamily (Liu et al. 2017). In the PB90 treatment of *S. bambusicola*, *MFS* gene expression also increased possibly because *S. bambusicola* cells were the first to produce a defense response, the *MFS* transporters was activated and involved in this process, possibly transporting the secondary metabolites to prevent PB90 invasion. The expression levels of *PKS*, *FAD*, and *MFS* genes significantly decreased when NO inhibitor and CAT were added compared with their corresponding levels when PB90 was added. When only NO inhibitor or CAT was added, the expression levels of *PKS*, *FAD*, and *MFS* genes slightly decreased. These results further confirmed that NO and H_2_O_2_ were involved in the regulation of hypocrellin produced by *S. bambusicola* and activated by PB90, revealing a special interaction between NO and H_2_O_2_ in mediating PB90-stimulated hypocrellin synthesis. NO and H_2_O_2_ signaling pathways can be involved in the signal transduction of flavonoid synthesis in cells by UV light, these pathways can also participate in the signal transduction of hypericin synthesis in *H. perforatum* through thermal shock (Simic et al. 2015; Lazzara et al. 2017). However, hypocrellin biosynthesis induced by *Aspergillum niger* crude polysaccharide extract can be inhibited by cPITO (Du et al. 2015), and hypericin synthesis in hypericum cells induced by PB90 can be blocked by CAT, which is mainly affected by NADPH oxidation-mediated H_2_O_2_ signaling (Xu et al. 2011). Walker et al. (2002) showed that jasmonic acid (JA) increases hypericin production in the cell culture of *H. perforatum*, whereas SA and fungal elicitors *Phytophthora cinnamoni* do not cause any stimulating effects. These results suggested that the response of an induced cell might vary in different cell lines and under various culture conditions. For NO and H_2_O_2_, a detailed signal transduction pathway mediated by PB90-induced secondary metabolite synthesis should be further studied.

The cellular defense response is mediated by complex signaling networks (Tani and Funato 2018). Our results showed that PB90 was a good method to increase the yield of hypocrellin in a very short period culture of *S. bambusicola*, the research group studied the reason of improving the synthesis of hypocrellin. After joining PB90 induction culture, a large number of the main active substance hypocrellin synthesis from *S. bambusicola*, biomass decreased, stability of vesicles were reduced, phenols and flavonoids were synthesized, the activities of PAL and CHI in the phenylpropanoid/flavonoid pathway were improved. This indicated that PB90 not only activated the massive synthesis of hypocrellin, but also changed the accumulation of phenols compounds with high antioxidant activity. Pb90 activated the phenylalanine/flavonoid pathway to stimulate the synthesis of hypocrellin. The study also found that PB90 treatment could not only directly induce the synthesis and accumulation of NO and H_2_O_2_ in *S. bambusicola* cells, but also that NO and H_2_O_2_ in the cells under PB90 treatment could improve their signal levels through interaction. Synthesis of hypocrellin was related to genes of *PKS*, *FAD* and *MFS*, and it was further confirmed at the molecular level that NO and H_2_O_2_ mediated the special signal interaction of hypocrellin induced by PB90. To investigate the relationship between hypocrellin, total phenols, total flavonoids, some enzymes, signaling molecules and gene expression of cells induced by PB90, in order to elucidation the regulatory mechanism of PB90 on the hypocrellin synthesis by *S. bambusicola*. This indicated that the secondary metabolites produced by *S. bambusicola* in liquid culture could be treated with PB90, which could be used as a production route for rapidly increasing the main pharmacological activity of hypocrellin. PB90 treatment shows a promising approach for studying the biosynthetic pathways of fungal secondary metabolites. Therefore, we suggest that this study also play an exemplary role in the study of the application of PB90 in the protection and sustainable utilization of other medicinal fungi resources.

## Supplementary information

**Additional file 1.** Response mechanism of hypocrellin colorants biosynthesis by *Shiraia bambusicola* to elicitor PB90.

## Data Availability

We conducted experiments and data were generated. All data is shown in Figures and Tables within the article.

## Abbreviations

H_2_O_2_: hydrogen peroxide
NO: nitric oxide
cPITO: carboxy-2-phenyl-4,4,5,5-tetramethylimidazoline-1-oxyl-3-oxide
SOD: superoxide dismutase
CAT: catalase
TEM: transmission electron microscopy
SEM: scanning electron microscopy
PAL: phenylalanine ammonia-lyase
CHI: chalcone isomerase
SA: salicylic acid
ROS: reactive oxygen species
SNP: sodium nitroprusside
*PKS*: polyketide synthase
*FAD*: FAD/FMN-dependent oxidoreductase
*MFS*: major facilitator superfamily transporter
PDA: potato dextrose agar
