# Supplementary material for: Response mechanism of hypocrellin colorants biosynthesis by Shiraia bambusicola to elicitor PB90
Source: AMB Express. 2019 Sep 14;9:146. doi: 10.1186/s13568-019-0867-5 (PMC6745040; doi:10.1186/s13568-019-0867-5)
Supplement: Supplementary file 1 — Additional file 1. Response mechanism of hypocrellin colorants biosynthesis by Shiraia bambusicola to elicitor PB90. [file 13568_2019_867_MOESM1_ESM.doc]

**Additional Information**

Analysis of related gene expression

Primer probes design. The method of Zhao et al. (2016) and Lei et al. (2017) were improved slightly. According to the known genome sequence of *S. bambusicola* from the gene bank (<http://www.ncbi.nlm.nih.gov/nuccore/>axzn00000000), the gene sequence was compared and analyzed by Bio Edit software and Blast analysis was conducted to determine the selected regions were highly conserved and specific. Primers were designed by Primer Premier and Beacon Designer. The designed primer is sent to the biotechnology company for synthesis. The polyketide synthase (*PKS*), FAD/FMN-dependent oxidoreductase (*FAD*) and major facilitator superfamily transporter (*MFS*) were selected (Table S1) to determine and analyze the expression differences of related genes before and after the effects of PB90.

Table S1 Real-time fluorescence quantitative primer sequences for the gene transcription level analysis.

F: forward primer, R: reverse primer

| Genes | Primer sequences（5′-3′） |
| --- | --- |
| *PKS* | F:TGCTGAGGTAGCAGTCAAGC  R:TTATGCTACGGTCGTCGCTC |
| *FAD* | F:ACGAGGTTTGGCATCGTCAT  R:ACGAATATGCCCGACTCCAC |
| *MFS* | F:CAGAAAGCAAGGCTACGGGA  R:TCACCAAATCGCCGAAGGAA |
| 18S | F:ACGCAGCGAAATGCGATAAG  R:CAAATTGTGCTGCGCTCCAA |

Zhao N, Lin X, Qi SS, Luo ZM, Chen SL, Yan SZ. De novo transcriptome assembly in *Shiraia bambusicola* to investigate putative genes involved in the biosynthesis of hypocrellin A. International Journal of Molecular Sciences, 2016, 17, 311; doi:10.3390/ijms17030311

Lei XY, Zhang MY, Ma YJ, Wang JW. Transcriptomic responses involved in enhanced production of hypocrellin A by addition of Triton X‑100 in submerged cultures of *Shiraia bambusicola.* J Ind Microbiol Biotechnol, 2017, DOI 10.1007/s10295-017-1965-5

Fig. S1. Effects of PB90 on biomass. Tree-day-old cells treated with 5 nmol/L PB90 were harvested as the time indicated in the figure. The control received vehicle solvent only. The results shown are the average of five independent experiments. Bars represented standard errors.
